# The Prevalence of Sleep-Disordered Breathing in Children With Neuromuscular Diseases

**DOI:** 10.7759/cureus.109251

**Published:** 2026-05-20

**Authors:** Amal R Al-Naimi, Haneen M Toma, Khalid Ibrahim, Mohammed Abushahin, Ahmed Abushahin

**Affiliations:** 1 Pediatric Pulmonology, Sidra Medicine, Doha, QAT; 2 Pediatric Neurology, Sidra Medicine, Doha, QAT; 3 Pediatrics, University of Lancashire, Preston, GBR

**Keywords:** central hypoventilation, neuromuscular disease, obstructive sleep apnea, polysomnography, sleep disordered breathing

## Abstract

Introduction

Sleep-disordered breathing (SDB) is common in children with neuromuscular disease (NMD). The occurrence and type of SDB depend on the specific NMD and the severity of neuromuscular weakness. SDB can lead to serious health problems and increase the risk in these patients. This study aimed to determine the prevalence of SDB in patients with NMD.

Methods

A retrospective chart review was performed of patients with a confirmed diagnosis of NMD who underwent overnight diagnostic polysomnography (PSG) at Sidra Medicine in Doha, Qatar. Clinical and PSG data were collected and summarized.

Results

We identified 53 patients with NMD (28 males, 52.8%) who underwent overnight PSG, with a mean age of 8.49 years (range: 0.25-18.7 years). The prevalence of obstructive sleep apnea (OSA) was 45.28% (24 of 53), with severity levels distributed as follows: mild (58%), moderate (33.3%), and severe (8.3%). The mean obstructive apnea-hypopnea index (OAHI) was 2.4 events per hour.

Conclusions

This study demonstrates a high prevalence of SDB among our cohort of patients with NMD. The predominant phenotype identified was OSA.

## Introduction

Neuromuscular diseases (NMDs) are a diverse group of disorders that affect the muscles, nerves, or neuromuscular junctions. The most common neuromuscular diseases in children include Duchenne muscular dystrophy (DMD), spinal muscular atrophy (SMA), and congenital muscular disorders (CMD) [1]. Respiratory muscles are universally affected in NMDs. The severity and progression of the disease determine respiratory outcomes. Due to respiratory muscle weakness, patients with NMD are susceptible to recurrent chest infections, aspiration, atelectasis, sleep disordered breathing (SDB), and progression to respiratory failure [2,3].

During sleep, many physiological changes occur, including decreased muscle tone, especially in the pharyngeal dilator muscles, which leads to increased upper airway resistance. There is also a diminished ventilatory response to hypoxia and hypercapnia. As a result, arterial carbon dioxide tension (PaCO₂) can rise by 4-8 mmHg, and arterial oxygen tension (PaO₂) can decrease by 3-10 mmHg [4]. These changes are more pronounced during REM sleep because of generalized atonia. While healthy children are usually able to compensate for these shifts and maintain adequate gas exchange, patients with NMD may struggle due to underlying respiratory muscle weakness, altered respiratory drive, chest wall deformities, and craniofacial malformations. These factors can lead to hypoventilation and sleep-related respiratory failure, which may eventually cause respiratory failure even during wakefulness [4]. 

Routine respiratory assessment is crucial for patients with NMDs and should include spirometry, lung volume measurements, maximal inspiratory and expiratory pressures, and peak cough flow. Because of the increased risk of SDB in this group, overnight polysomnography (PSG) is also recommended [3]. The timing of PSG should depend on the severity and progression of the disease. For instance, in patients with DMD, PSG is recommended once they become wheelchair-dependent or when clinical symptoms indicate sleep-related breathing problems [5]. SDB in NMD patients can present as sleep-related hypoventilation, nocturnal hypoxemia, obstructive sleep apnea (OSA), central sleep apnea (CSA), and/or abnormal control of breathing [6].

Management of SDB in patients with NMD depends on the type and severity of the underlying condition. In progressive NMDs, chronic respiratory failure is a common outcome. Early identification of nocturnal hypoventilation and impending respiratory failure is critical because it allows for timely intervention and better preparation for procedures requiring sedation, such as surgery or radiological imaging. Non-invasive ventilation (NIV) is widely used in NMD patients and has been shown to improve survival and quality of life [7]. The primary objective of our study was to determine the prevalence of SDB in patients with NMD.

## Materials and methods

Study design

A retrospective, cross-sectional study was conducted on children with NMD who were referred for overnight PSG at Sidra Hospital from September 1, 2019, to March 30, 2025.

Patient recruitment

Patients were identified from the electronic sleep lab software based on a diagnosis of NMD. All patients aged 18 years or younger were included. Patients with poor sleep efficiency (<40%) or incomplete data were excluded.

Data collection

The following clinical data were collected from the electronic health system: age, gender, weight (BMI), symptoms (snoring, witnessed sleep apnea), comorbidities (gastroesophageal reflux, aspiration syndrome, asthma, allergic rhinitis, scoliosis), cardiac disease, pulmonary hypertension, reported adenoid/tonsil hypertrophy, adenotonsillectomy, persistent OSA after adenotonsillectomy, and reported airway malacia.

PSG data were obtained from the sleep lab software. The overnight PSG was performed using the Philips Respironics Alice 6 PSG system with Sleepware G3. PSG data included sleep efficiency, sleep stages, sleep body positions, as well as the apnea-hypopnea index (AHI), REM-related apnea-hypopnea index (REM-AHI), obstructive apnea-hypopnea index (OAHI), central apnea-hypopnea index (CAHI), central apnea index (CAI), average oxygen saturation (SpO₂), lowest SpO₂, percentage of total sleep time (TST) spent with SpO₂ <90%, and average end-tidal CO₂ (EtCO₂).

All events and parameters were scored according to the American Academy of Sleep Medicine (AASM) Staging and Scoring Manual, Version 2.5, 2018. OSA was defined as an OAHI ≥1.5 events per hour. OSA severity was classified as mild if OAHI was between 1.5 and 4.99 events/hour, moderate if between 5 and 9.99 events/hour, and severe if ≥10 events/hour. CSA was defined as a CAI ≥5 events/hour. REM-related OSA was defined as a REM OAHI to NREM OAHI ratio of ≥2, while positional OSA was defined as a supine OAHI to non-supine OAHI ratio of ≥2.

Statistical analysis

Demographic and clinical characteristics were summarized as mean and standard deviation (SD) for normally distributed continuous variables and median (range) for skewed continuous variables.

## Results

A total of 53 patients with NMD (28 males and 25 females) were included. The mean age of the cohort was 8.49 years (range: 0.25-18.7), and the mean BMI was 16.6 (range: 11-26.25). Only a few patients were symptomatic; eight reported snoring, and two patients reported witnessed sleep apnea. Eighteen patients had a history of aspiration, and almost half had scoliosis. The most common diagnoses were DMD (11/53, 20.8%), congenital merosin dystrophy LAMA2 (11/53, 20.8%), and limb-girdle muscular dystrophy (LGMD) (7/53, 13.2%). Detailed demographic and clinical data are presented in Table 1.

**Table 1:** Demographic and clinical data of the NMD patients (n=53) NMD: neuromuscular disease; BMI: body mass index; GERD: gastroesophageal reflux disease; SMA: spinal muscular atrophy; LAMA2: laminin subunit a2 gene

Characteristics	Values
Age, years, median (range)	8.49 (0.25-18.7)
Male gender, n (%)	28/53 (52.8%)
BMI, Kg/m^2^,median (range)	16.6 (11-26.25)
BMI centile, median (range)	42.2 (0-98)
Duchenne muscular dystrophy (DMD), n (%)	11 (20.8%)
Congenital merosin deficiency LAMA2 , n (%)	11 (20.8%)
Limb-girdle muscular dystrophy (LGMD), n (%)	7 (13.2%)
SMA type 1 and 2, n (%)	7 (13.2%)
Congenital myopathy, n (%)	7 (13.2.%)
Congental hypotonia, n (%)	6 (11.3%)
Congenital myasthenia, n (%)	4 (7.5%)
History of snoring, n (%)	8 (15%)
History of witnessed sleep apnea, n (%)	2 (3.7%)
History of GERD, n (%)	5 (9.4%)
History of aspiration, n (%)	18 (33.9%)
Scoliosis, n (%)	24 (45.2%)

The mean (range) sleep efficiency was 76.4% (45%-97%). The mean (range) sleep latency was 39.4 (1-244) minutes, and the mean (range) REM latency was 112.25 (6-295) minutes. The mean (range) wakefulness after sleep onset was 76.8 (4.5-271.5) minutes. Sleep architecture was characterized as follows: the mean (range) REM percentage of total sleep time was 21% (4.5-36.3). The mean N1 percentage was 5.2% (0.3-13.3), the mean N2 percentage was 47.54% (25-67), and the mean N3 percentage was 24.37% (13-47). The arousal index had a mean (range) of 8.39 (0.7-24). The AHI had a mean (range) of 3.98 (0.1-28.6) events/hour; the OAHI was 2.4 (0-12.24) events/hour; the CAHI was 1.51 (0-21.9) events/hour; and the CAI was 0.45 (0-4.1) events/hour. The REM AHI was 12.72 (0-111) events/hour; the REM OAHI was 9 (0-40.5) events/hour; and the NREM OAHI was 1.83 (0-24) events/hour. The mean oxygen desaturation index (ODI) was 6.97 (0-55). The mean oxygen saturation (SpO₂) was 96% (90-99), with a mean minimum SpO₂ of 87% (30%-95%). The median percentage of time spent with SpO₂ < 90% was 1.43% (0-26). Peak EtCO₂ was 45 mmHg (29-60 mmHg), with only 11 patients demonstrating a peak EtCO₂ ≥50 mmHg (Table 2).

**Table 2:** Polysomnography results of NMD patients (n=53) NMD: neuromuscular disease; WASO: wake after sleep onset; TST: total sleep time; AHI: apnea-hypopnea index; OAHI: obstructive apnea-hypopnea index; CAHI: central apnea-hypopnea index; CAI: central apnea index; OSA: obstructive sleep apnea; SPO_2_: oxygen saturation by pulse oximetry; ODI_3_: oxygen desaturation index (³3% drops in SPO_2_ per hour of sleep); ETCO_2_: end-tidal carbon dioxide

Parameter	Values
Sleep efficiency, %, median (range)	76.4 (45-97)
WASO, minutes, median (range)	76.8 (4.5-271.5)
Sleep latency, minutes, median (range)	39.4 (1-244)
REM latency, minutes, median (range)	112.25 (6-295)
N1% of TST, median (range)	5.2 (0.3-13.3)
N2% of TST, median (range)	47.54 (25-67)
N3% of TST, median (range)	24.3 (13-47)
REM% of TST, median (range)	21 (4.5-36.3)
Snoring recorded during polysomnography %	11
AHI, events/hour, median (range)	3.98 (0.1-28.6)
OAHI, events/hour, median (range)	2.4 (0-12)
REM AHI, events/hour, median (range)	12.7 (0-111)
REM OAHI, events/hour, median (range)	9 (0-40.5)
NREM OAHI, events/hour, median (range)	1.83 (0-24)
CAHI, events/hour, median (range)	1.5 (0-21.9)
CAI, events/hour, median (range)	0.45 (0-4.1)
OSA, n (%)	24/53 (45.28%)
Mild OSA, n (%)	14/24 (58.3%)
Moderate OSA, n (%)	8/24 (33.3%)
Severe OSA, n (%)	2/24 (8.3%)
Average SPO_2_^,^% median (range)	96 (90-99)
SPO_2 _nadir, %, median (range)	87 (30-95)
% of TST with SpO_2_<90%, median (range)	1.4 (0-26)
ODI_3_, events/hour, median (range)	6.9 (0-55)
Peak ETCO_2_, mmHg, median (range)	45 (29-60)
Arousal index, events/hour, median (range)	8.3 (0.7 -24)

OSA was diagnosed in 24 patients (45.28%), with the majority exhibiting mild OSA. Of those diagnosed, 58.3% had mild OSA, 33.3% had moderate OSA, and 8.3% had severe OSA. Mild to moderate OSA was observed in seven patients (63.6%) with DMD, with a mean age of 11.48 years (range: 6.08-16.83), and in five patients (45.5%) with congenital merosin deficiency due to LAMA2 mutations, with a mean age of 5.4 years (range: 0.58-14.25). Most patients with spinal muscular atrophy (SMA 1,2) demonstrated normal polysomnography or mild OSA, likely reflecting the effects of post-gene therapy intervention. Positional OSA (POSA) was observed in 18 patients (33.9%), while REM-related OSA was identified in 35 patients (66%). Among those with POSA, 14 out of 18 patients (77.7%) also had REM-related OSA. Conversely, only 14 out of 35 patients (40%) with REM-related OSA had POSA. No significant central apnea was observed. All assessments were performed using spot-check EtCO₂ measurements; therefore, nocturnal hypoventilation could not be confirmed.

## Discussion

Our study revealed a high prevalence of OSA among patients with NMD, with an estimated rate of 45.28%, similar to findings reported in comparable groups. Labanowski et al. studied 60 patients, both adults and children (33 males, 27 females), with various neuromuscular conditions and reported a 42% prevalence of SDB [8]. Similarly, Withers et al. assessed 66 children aged 6.0-16.7 years (median age: 11.5), of whom 73% were male. The cohort included 27 with DMD, 18 with SMA, eight with congenital muscular dystrophy, and 12 with congenital myopathy. Ten patients used NIV during the entire PSG. OSA was identified in 32% (n=21), hypoventilation in 18% (n=12), and CSA in 14% (n=9) [9].

In another study, Kalyoncu et al. evaluated 174 patients with a median age of 10 years (interquartile range: 5.1-13.5), of whom 71.3% were male. Of these, 54 (31%) had DMD, 23 (13.2%) had SMA, and 64 (36.8%) had other myopathies. OSA was present in 48.3%, with 56 patients (32.2%) classified as having mild OSA, 12 (6.9%) as moderate, and 16 (9.2%) as severe [10]. Most of our patients had mild OSA. The prevalence and severity of SDB vary depending on the specific NMD diagnosis and the timing of PSG, as some conditions tend to worsen with age. Also, all our patients with SMA had received gene therapy before undergoing PSG.

REM-related OSA was identified in 35 patients (66%), likely due to the characteristic physiological changes that occur during REM sleep. In NMD patients, SDB usually begins during REM, later extending into NREM sleep as the disease progresses, and may eventually result in daytime (diurnal) respiratory failure [11]. The majority of our patients had mild OSA and REM-related OSA, which may reflect early diagnosis and detection of sleep-disordered breathing. Symptoms were uncommon in our NMD patients, emphasizing the importance of screening with PSG. PSG remains the gold standard for assessing SDB in children [12]. Although sleep architecture can be disrupted in patients with NMD, typically resulting in decreased sleep efficiency, frequent arousals, and reduced REM sleep, these disturbances were not common in our patients [13].

Our retrospective study has several limitations, including a small overall number of NMD patients as well as a limited sample size within each NMD subgroup. Nocturnal hypoventilation is common in NMD patients; however, we were unable to evaluate this due to the absence of continuous CO₂ data in the PSG recordings. Nonetheless, this study may help inform the design of a prospective study, especially since our NMD cohort continues to expand due to the high rate of consanguineous marriages.

## Conclusions

Our study demonstrates a high prevalence of SDB in patients with NMD, regardless of the presence of symptoms. These findings support recommendations to screen all individuals with NMD for sleep breathing disorders, especially before surgery. With the introduction of new disease-modifying therapies for NMD patients, the natural course of disease may be altered, leading to changes in pulmonary outcomes that may influence pulmonary evaluation.
